# ASA score is an independent predictor of 1-year outcome after moderate-to-severe traumatic brain injury

**DOI:** 10.1186/s13049-025-01338-x

**Published:** 2025-02-06

**Authors:** Olivia Kiwanuka, Philipp Lassarén, Alexander Fletcher-Sandersjöö, Charles Tatter, Jonathan Tjerkaski, David W. Nelson, Eric P. Thelin

**Affiliations:** 1 Department of Clinical Neuroscience, Karolinska Institutet, Stockholm, Sweden; 2 Department of Surgery, Södersjukhuset, Stockholm, Sweden; 3 Department of Neurosurgery, Karolinska University Hospital, Stockholm, Sweden; 4 Department of Radiology, Södersjukhuset, Stockholm, Sweden; 5 Department of Clinical Sciences, Danderyd’s Hospital, Karolinska Institutet, Stockholm, Sweden; 6 Section of Perioperative Medicine and Intensive Care, Department of Physiology and Pharmacology, Karolinska Institutet, Stockholm, Sweden; 7 Department of Perioperative Medicine and Intensive Care, Karolinska University Hospital, Stockholm, Sweden; 8 Medical Unit Neurology, Karolinska University Hospital, Stockholm, Sweden

**Keywords:** Moderate/severe TBI, ASA score, IMPACT, 1-year outcome, 90-day mortality

## Abstract

**Purpose:**

This study aimed to investigate whether incorporating pre-injury health status, measured by the American Society of Anesthesiologists (ASA) score, improves outcome prediction models for moderate-to-severe traumatic brain injury (msTBI) patients.

**Methods:**

We conducted a retrospective single-center study of msTBI patients (2005–2021). The primary outcome was 1-year Glasgow Outcome Scale (GOS, dichotomized as GOS1-3 (unfavorable) vs. 4–5 (favorable)), and secondary outcome was 90-day mortality. Logistic regression evaluated the contribution of ASA score to the International Mission for Prognosis and Clinical Trials in Traumatic Brain Injury (IMPACT) core + CT outcome prediction model incorporating age, admission GCS, pupillary reactivity, Marshall CT classification, hypoxia, hypotension, epidural hematoma, and subarachnoid hemorrhage.

**Results:**

Among the 720 adult patients that were included 51% had an unfavorable GOS at 1 year. The 90-day mortality was 19%. ASA score and TRISS were independently associated with both outcomes (*p* < 0.001). Incorporating the ASA score to our IMPACT model significantly enhanced its explanatory value of dichotomized GOS (35% vs. 32% variance explained, *p* < 0.001) and improved the model’s prognostic accuracy.

**Conclusion:**

In this retrospective single-center cohort study, we found that ASA score improves existing prognostic models for msTBI. Incorporating this simple comorbidity measure could enhance outcome prediction and support more personalized acute management. Future prospective studies are needed to validate these results.

**Supplementary Information:**

The online version contains supplementary material available at 10.1186/s13049-025-01338-x.

## Introduction

Traumatic Brain Injury (TBI) represents a significant global health concern [1]. While historically considered a condition primarily affecting the young, it is now increasingly impacting the elderly population [2]. Accurate prediction of outcomes in TBI cases is crucial, as it influences patient management, policymaking, resource allocation, and clinical trial design [3]. Current prognostic models, such as the Corticosteroid Randomization After Significant Head Injury (CRASH) and the International Mission for Prognosis and Analysis of Clinical Trials in TBI (IMPACT) models, utilize demographic, clinical, biochemical, and radiological variables to predict outcomes after TBI, such as Glasgow Outcome Scale (GOS) [4–6]. Despite these advancements, these models explain only about 25–40% of the variance in predicted outcomes, even when combined with serum protein biomarkers of brain injury, more granular imaging metrics at admission, and data from the intensive care unit period [7–12]. This suggests that much of the variance in these prediction models cannot be explained by the trauma and subsequent injuries alone, pointing to the potential influence of other factors not generally included in TBI studies.

The Trauma and Injury Severity Score (TRISS) is a well-established mortality prediction tool in trauma care, integrating anatomical and physiological parameters to estimate the probability of survival [13, 14]. The anatomical component is derived from the Injury Severity Score (ISS), which calculates a total based on the squared Abbreviated Injury Scale (AIS) scores of the three most severely injured body regions. The physiological component is based on the Revised Trauma Score (RTS). While TRISS is well-established in general trauma [15] its performance in specific subgroups is fully studied. In our previous study, TRISS demonstrated poor correlation with mortality in complicated mild TBI (mTBI) [16], but it holds promise as a prognostic tool in moderate-to-severe (ms-) TBI, as supported by other studies [17].

Age is frequently included in these models and is widely recognized as a negative predictive factor due to the increased physiological vulnerability and the higher likelihood of pre-existing health conditions in older adults [2, 18]. The presence of comorbidities, a prevalent issue among the elderly, further complicates management, recovery, and prognosis. However, the impact of comorbidities on TBI outcomes has been challenging to quantify, partly due to the varied ways comorbidities are defined and measured across studies [19].

The American Society of Anesthesiologists (ASA) score, a globally recognized tool for preoperative assessment, categorizes patients based on their overall health status and pre-existing comorbidities [20]. It has demonstrated reliability in assessing comorbidity burden and has been identified as an independent predictor of in-hospital and 30-day mortality following trauma [21, 22]. Studies assessing ASA score in TBI are scarce [18], especially in the more severe spectrum. Our previous research in mild complicated TBI has found ASA-score to be the strongest predictor of long-term health related quality of life after TBI [23] and an independent predictor of 90-day mortality after mild TBI [24], highlighting its potential as a valuable tool for outcome prediction in TBI patients with milder injuries.

This study seeks to explore further the prognostic impact of comorbidities, as quantified by the ASA score, on the long-term outcomes following msTBI. Understanding these long-term outcomes is crucial for developing tailored treatment plans, informing patients and families about prognosis, and guiding healthcare policies to improve care for TBI patients.

## Methods

### Study design and ethical approval

This retrospective, single-center study received approval from the Swedish Ethical Review Authority (Dnr: 2019–04476 with amendments 2022-06135-02 and 2023-02224-02). The primary outcome was the 1-year Glasgow Outcome Scale (GOS), and a secondary outcome of 90-day mortality. The Swedish Ethical Review Authority waivered the need for informed consent.

### Study population

The study included adults (aged ≥ 15 years) admitted to the neurosurgical department at Karolinska University Hospital, the region’s trauma center, from 2005 to 2021. Exclusion criteria were patients lacking essential data such as ASA-score or admission imaging, or those transferred from other hospitals for convalescent care.

### Data collection

The TBI-registry at Karolinska University Hospital, and the Swedish trauma registry (SweTrau) were used for data collection. The TBI registry includes all TBI-patients treated by the neurosurgical department. SweTrau is based on the revised Utstein template [25]. Patients with TBI were identified from the local trauma database including all patients in Region Stockholm requiring neurosurgical management or monitoring for their TBI. Collected demographic data from medical records included the first recorded measurements of Glasgow Coma Scale (GCS), systolic blood pressure (SBP) (hypotension if < 90mmHg at the scene of accident), pupillary reaction (defined as normal, one pupil without light reflex or if bilaterally absent, at hospital admission), oxygen saturation (hypoxia if < 90% saturation at the scene of accident).

### Assessment tools and scoring systems

Multiple scoring systems and models were employed. Accredited and independent professionals performed the scoring of AIS and ASA scores. The AIS scoring was based on the assessment of retrospective clinical and radiological findings per established guidelines [26]. Regular quality control of AIS scoring is performed through randomized validation checks. In cases of significant discrepancies, a panel of multiple certified professionals conducts a comprehensive review. The pre-injury ASA score was either established by the treating physician at the time of care or retrospectively determined through assessment of the medical record.

### ASA-score

The ASA score assesses a patient’s preoperative health status, ranging from ASA I (“healthy”) to ASA VI (“brain-dead, organ donation”) [20].

### Injury severity scores and polytrauma

AIS is an anatomical trauma score primarily based on radiological findings [27, 28]. Injuries are graded between 1 (minor) to 6 (maximal, fatal), and divided into eight different anatomical regions; head, face, neck, thorax, abdomen, spine, upper extremities, lower extremities, and external. Polytrauma is here defined as significant traumatic injuries (AIS ≥ 3), in 3 or more body regions [29]. The injury severity score (ISS) is an anatomical scoring system that provides an overall score for patients with multiple injuries. Each injury is assigned an AIS allocated to one of six body regions. The highest AIS score in each body region is used and ISS ranges from 0 to 75. The new injury seerity score (NISS) considers the three most severe AIS scores, regardless of the body region. The scores are squared (e.g., 3 becomes 9) and then summed with no upper limit.

### Trauma and Injury Severity score

The Trauma and Injury Severity Score (TRISS) is a widely recognized method used to predict the likelihood of survival following a traumatic injury. It combines the Revised Trauma Score (RTS), which includes physiological parameters like respiratory rate, systolic blood pressure, and GCS, with the ISS and the patient’s age [14, 30]. Its complete formula is detailed in Supplementary Table 1.

### IMPACT

IMPACT is a prognostic model that consolidates data from eight randomized controlled trials and three observational studies spanning 1984 to 1997 [5, 6]. We used the parameters of the core + CT model that include age, motor score, pupillary reactivity, hypoxia, hypotension, Marshall CT classification, and presence of epidural hematoma (EDH) and traumatic subarachnoid hemorrhage (trSAH).

### Glasgow outcome scale

GOS is a 5-level scale that was developed to assess global disability after TBI: GOS 1 = dead, GOS 2 = vegetative state, GOS 3 = severe, dependent state, GOS 4 = moderately recovered, independent state, and GOS 5 = good recovery [31]. GOS was assessed clinically at 3–6 months post-trauma at outpatient clinical visits, and at around 12 months via questionnaire. GOS was further dichotomized into *favorable* (GOS 4–5) and *unfavorable* (GOS 1–3) outcome.

### Statistical analysis

All data was analysed using R [32] through the visual interface R-studio (v. 2022.07.2 Build 576, PBC, USA). Normality was tested with the Shapiro-Wilk test. Results are presented as median with interquartile range for continuous data, and n (%) for nominal data, if not stated otherwise. Baseline characteristics were assessed using the Mann-Whitney U test for quantitative variables, chi-squared test for categorical variables with expected count of at least 5, and Fischer’s exact test for categorical variables with expected count of less than 5, and ordered logit for ordinal variables. The significance level was set to 0.05. Logistic regression analyses, both univariable and multivariable, assessed the impact of various factors on the 12-month GOS.

### Missing data

To address the missing data within our dataset, we employed the technique of multiple imputation (MI), performing seven separate imputations as recommended by established statistical literature and the IMPACT research group [33, 34]. The median was computed for p-values [35], and an average is presented for the remaining variables.

## Results

### Demographics

**Fig. 1:**
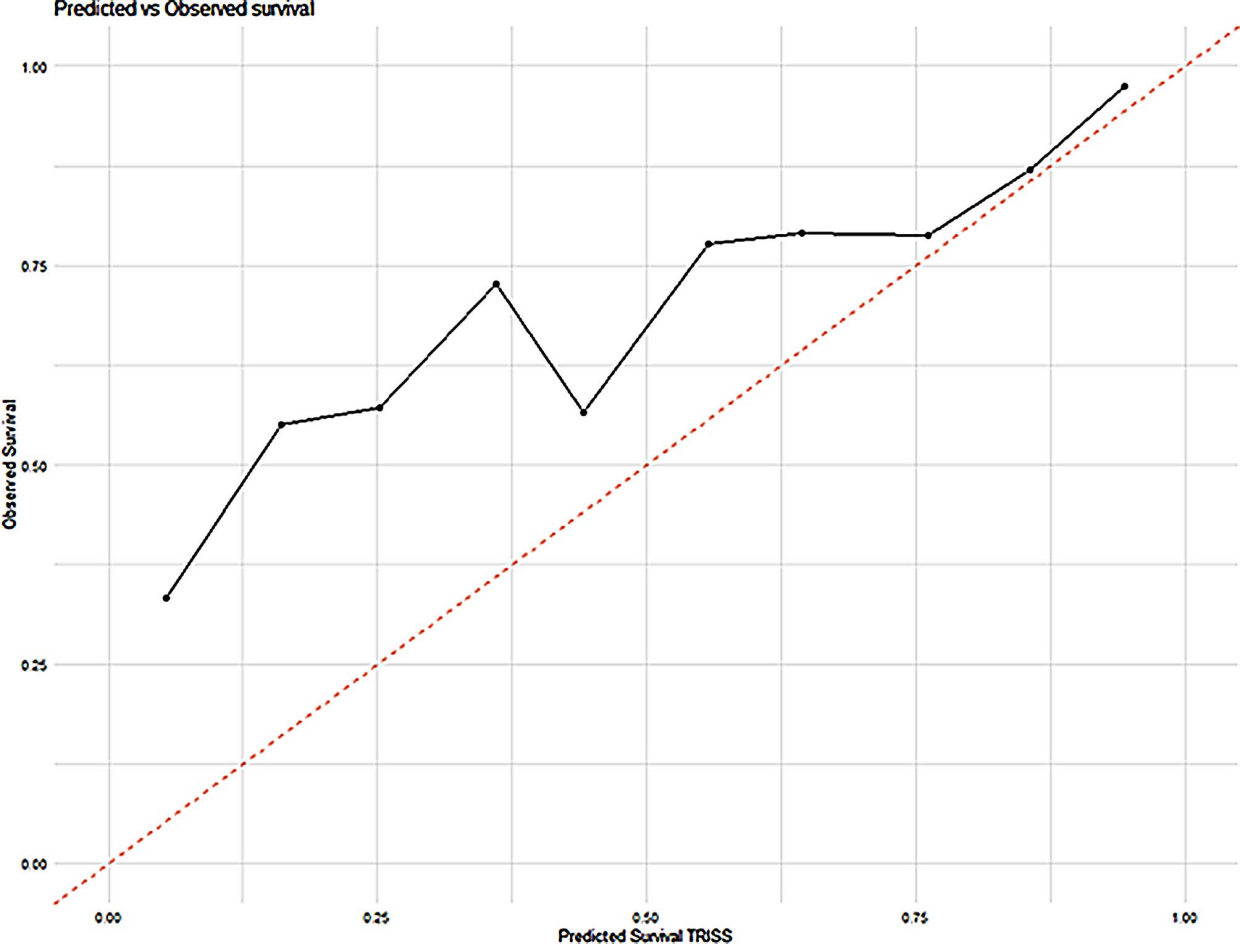
*TRISS*. The figure compares the predicted versus observed survival rates with TRISS at 1-year (GOS 1). The x-axis displays the predicted survival probability based on the TRISS, while the y-axis shows the observed survival rate. The diagonal dashed line represents the line of perfect prediction where the predicted probabilities match the observed outcomes exactly. Points above this line indicate better than expected survival rates, while points below the line suggest lower than expected survival. GOS: Glasgow Outcome Scale, TRISS: Trauma and Injury Severity Score

A total of 720 patients with msTBI were included in our analysis (Supplementary Fig. 1). The demographics, outlined in Table 1, revealed a male predominance (74.3%) with a median age of 51 years. Half of the cohort (49%) were healthy (ASA score 1), whereas 23% had a severe systemic disease (ASA score of 3 or 4). The most common mechanisms of injury were low-energy falls (33.4%), followed by high-energy falls (21.7%). The patient cohort was severely injured with a median ISS of 25, with a third (34.8%) of the patients experiencing polytrauma. EDH and trSAH were noted in 50% and 60% of cases, respectively. One-year outcomes ranged across the GOS spectrum with half of the cohort (49%) having a favorable outcome (GOS 4–5) after 12 months. A fifth of the cohort (20.0%) were deceased (GOS 1), of which the vast majority (19% of the cohort and 92% of the deceased) died within the first 90 days. TRISS suggested a median survival probability of 79%, slightly lower than the observed 90-day mortality (Fig. 1). The median length of hospital stay was 13 days, and follow-up post-trauma was conducted at a median of 345 days.

**Table 1:** Demographic characteristics

Parameter	Subcategory or units	Numerical median [IQR] or number (%)
**Pre-admission data**		
Age	Years	51 [31, 65]
Gender	Male	535 (74%)
ASA score	1. Healthy	356 (49%)
	2. Mild systemic disease	200 (28%)
	3. Severe systemic disease	151 (21%)
	4. Severe systemic disease with constant threat to life	13 (1.8%)
MOI	Traffic accident	128 (28%)
	Assault	7 (1.5%)
	Blunt trauma	63 (14%)
	Low energy fall	160 (34%)
	High energy fall	104 (22%)
	Other	2 (0.4%)
	Unknown	256
Polytrauma	Yes	250 (35%)
	Unknown	2
Alcohol	Present	240 (36%)
	Unknown	50
**Admission data**		
GCS	3–8	529 (73%)
	9–12	191 (27%)
TRISS	points	79 [54, 91]
	*Unknown*	281
NISS	points	41 [27, 50]
	Unknown	241
Hypoxia	Yes	89 (17%)
	Unknown	209
Hypotension	Yes	28 (3.9%)
	Unknown	9
Pupillary reaction	Responsive	518 (76%)
	Unilateral unresponsive	53 (7.7%)
	Bilateral unresponsive	115 (17%)
	Unknown	34
**Radiology**		
trSDH	Yes	380 (60%)
	Unknown	82
EDH	Yes	318 (50%)
	Unknown	82
Marshall classification	*II*	246 (34%)
	*III*	112 (16%)
	*IV*	21 (2.9%)
	*V*	63 (8.8%)
	*VI*	278 (39%)
**Outcome**		
Mortality 90-day	Yes	133 (19%)
	*Unknown*	4
Time to assessment	Days	345 [174, 516]
GOS 1 year	1. Death	144 (20%)
	2. Vegetative state	7 (1.0%)
	3. Severe disability	216 (30%)
	4. Moderate disability	240 (33%)
	5. Good recovery	113 (16%)
	4–5 Favorable outcome	353 (49%)

Demography of included patients with complete data. Results expressed in median and (IQR) as well as numeric values and (%). ASA: American Society of Anesthesiologists Classification; MOI: Mechanism of injury; GCS: Glasgow Coma Scale; NISS: New Injury Severity Score; TRISS: Trauma and Injury Severity Score; GOS: Glasgow Outcome Scale

### One-year outcome

**Fig. 2:**
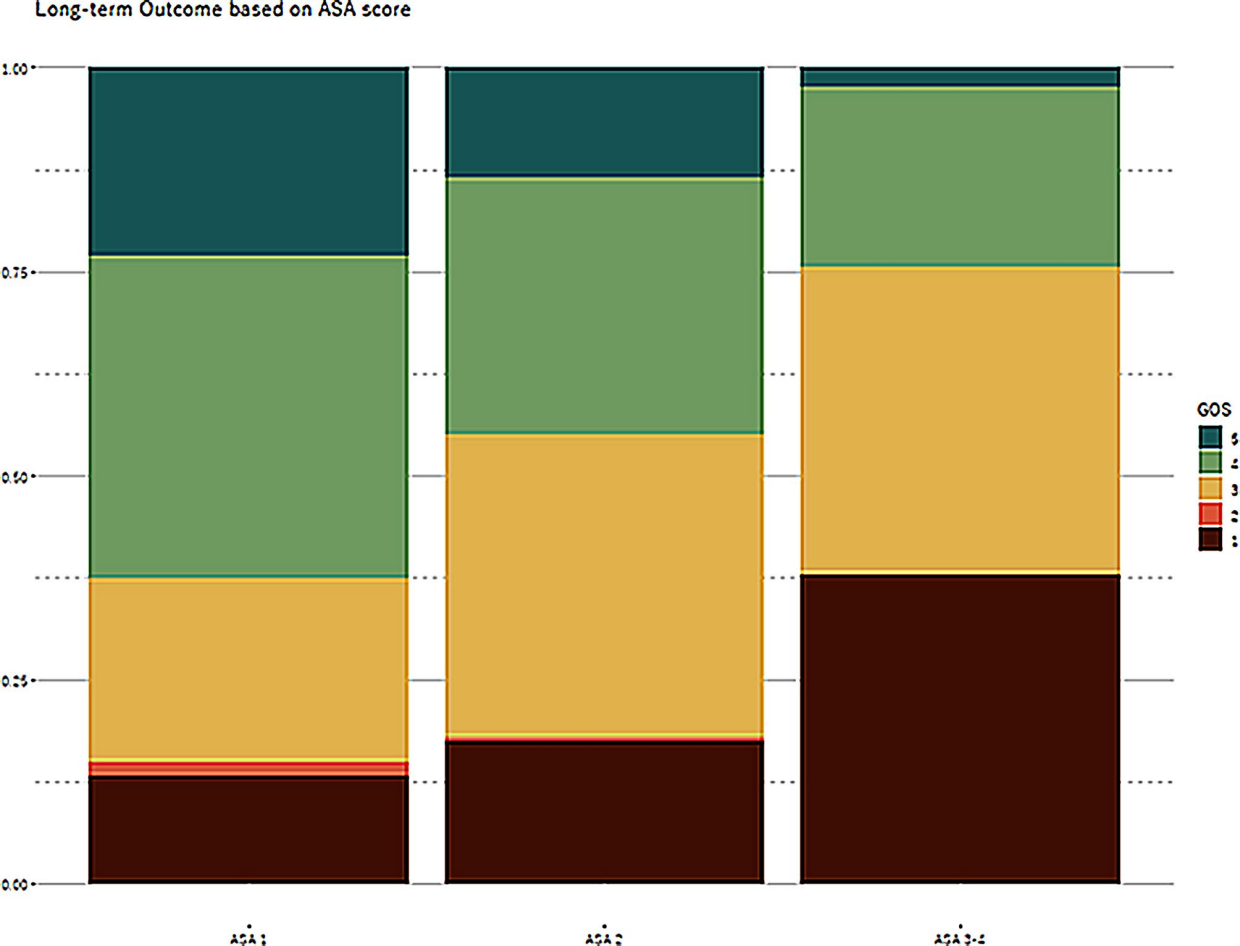
*GOS vs. ASA*. Illustration of the distribution of GOS one-year post-injury stratified by ASA score. Each bar represents the proportion of each outcome within the respective ASA category, conveying the relationship between pre-injury health status as measured by ASA scores and the long-term recovery trajectory of patients with traumatic brain injury. GOS: Glasgow Outcome Scale, ASA: American Society of Anesthesiologists Classification

Age, ASA score, GCS, TRISS, NISS, and pupillary reaction were all strong predictors of 1-year outcome (*p* < 0.001, Table 2). A higher ASA score was associated with lower GOS at 1 year (Fig. 2). ASA score of 4 was not significant, probably due to the low prevalence (*n* = 16, 2%) and was combined with ASA score 3 in the analysis. The Nagelkerke pseudo-R² values showed that TRISS was the parameter with the most significant contribution to the model, explaining 17% of the variance in outcomes.

**Table 2:** Parameters and GOS 1 year

		Dichotomized GOS				Non-dichotomized GOS			
Parameters	*p*-value	Nagelkerke pseudo R2	OR	AUC	AIC	Nagelkerke pseudo R2	AUC	AIC	*n*
**Pre-admission**									
Age	< 0.001 *	0.16	0.96 (0.95–0.97)	0.70	910	0.132	0.627	1909	720
Gender (Female)	0.253		0.82 (0.59–1.15)	0.52	1001	0.003	0.507	2002	720
ASA score		0.14		0.66	928	0.121	0.564	1921	720
*2*	< 0.001 *		0.49 (0.34–0.69)						
*3*	< 0.001 *		0.22 (0.14–0.33)						
*4*	0.968		0 (0-135065.07)						
Polytrauma (Yes)	0.453		1.13 (0.83–1.53)	0.51	998	0.001	0.5	1998	718
Alc (No)	< 0.001 *	0.04	0.47 (0.34–0.64)	0.59	911	0.031	0.5	1847	670
**Admission**									
GCS	< 0.001 *	0.05	1.13 (1.08–1.18)	0.60	975	0.043	0.594	1975	720
TRISS	< 0.001 *	0.17	1.03 (1.02–1.04)	0.72	550	0.171	0.684	1147	439
Hypoxia (Yes)	0.013 *	0.02	0.55 (0.34–0.88)	0.54	706	0.016	0.553	1416	511
Hypotension (Yes)	0.073		0.48 (0.2–1.04)	0.51	986	0.012	0.525	1973	711
Pupils		0.13		0.63	888	0.154	0.626	1806	686
*unilateral*	0.001 *		0.37 (0.2–0.67)						
*bilateral*	< 0.001 *		0.16 (0.1–0.26)						
**Radiology**									
trSAH	0.197		1.23 (0.9–1.69)	0.53	887	0.002	0.5	1764	638
EDH	0.342		1.16 (0.85–1.59)	0.52	888	0	0.5	1764	638

Summary of factors influencing the GOS at 1-year post-admission. Statistical significance was assessed using p-values, with effect sizes quantified by odds ratios (OR). AUC: Area Under the Curve; AIC: Akaike Information Criterion; ASA: American Society of Anesthesiologists Classification; GCS: Glasgow Coma Scale; TRISS: Trauma and Injury Severity Score; Alc: Alcohol; trSAH: traumatic subarachnoid hemorrhage; EDH: epidural hematoma

**Fig. 3:**
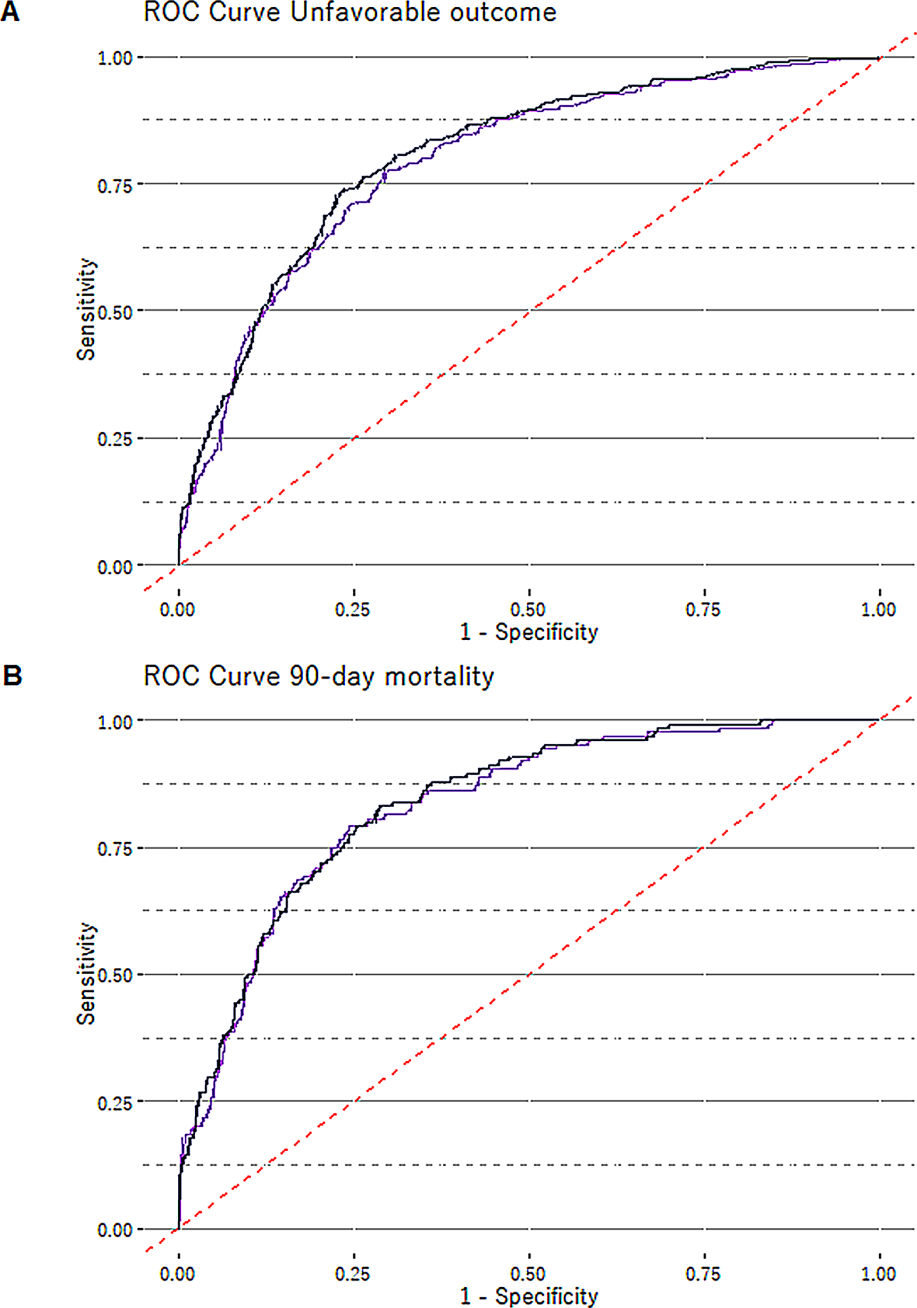
ROC curves evaluating the predictive performance of the IMPACT CT model alone (purple line) or enhanced with ASA score (blue line). Part A shows the ROC curve for unfavorable outcomes at 1 year (GOS 1–3), and Part B for 90-day mortality. The diagonal line from the bottom left to the top right serves as a reference indicating the performance of a non-discriminatory model; points above this line reflect a model with better predictive ability. ROC: Receiver Operating Characteristic, IMPACT: International Mission for Prognosis and Analysis of Clinical Trials in TBI, GOS: Glasgow Outcome Scale, ASA: American Society of Anesthesiologists Classification

Our IMPACT model, a combination of age, GCS, pupillary reaction, hypoxia, hypotension, and the radiological parameters Marshall CT classification, epidural hematoma and subarachnoid hemorrhage, had a pseudo-R² of 0.32 (Table 3). Adding ASA score significantly improved the model (*p* < 0.001), the explanatory value increasing to 35%, with a slight increase in the area under the curve (AUC), and decrease of the Akaike information criterion (AIC) (Fig. 3).

**Table 3:** ASA and TRISS in multivariable analysis with IMPACT extended variables

Parameters	*p*-value	Nagelkerke pseudo *R*^2^	AUC	AIC	*n*
IMPACT CT		0.321	0.793	822	720
IMPACT CT + ASA score	< 0.001 *	0.351	0.805	806	720
IMPACT CT + TRISS	0.014*	0.344	0.803	807	720

IMPACT core + CT model consists of age, motor score, pupillary reactivity, hypoxia, hypotension, Marshall classification, and occurrence of epidural hematoma and traumatic subarachnoid hemorrhage at hospital admission. Nagelkerke’s pseudo-R2 values are from multivariable regression models, where a value of 1 would fully predict unfavorable versus favorable outcome (GOS 1–3 versus 4–5). ASA score and TRISS significantly added independent information to the model, described by the p-values. Missing data was imputated to obtain a sample of 720. IMPACT: International Mission for Prognosis and Analysis of Clinical Trials in TBI; ASA: American Society of Anesthesiologists Classification; TRISS: Trauma and Injury Severity Score; AUC: Area Under the Curve; AIC: Akaike Information Criterion

### 90-day mortality

**Fig. 4:**
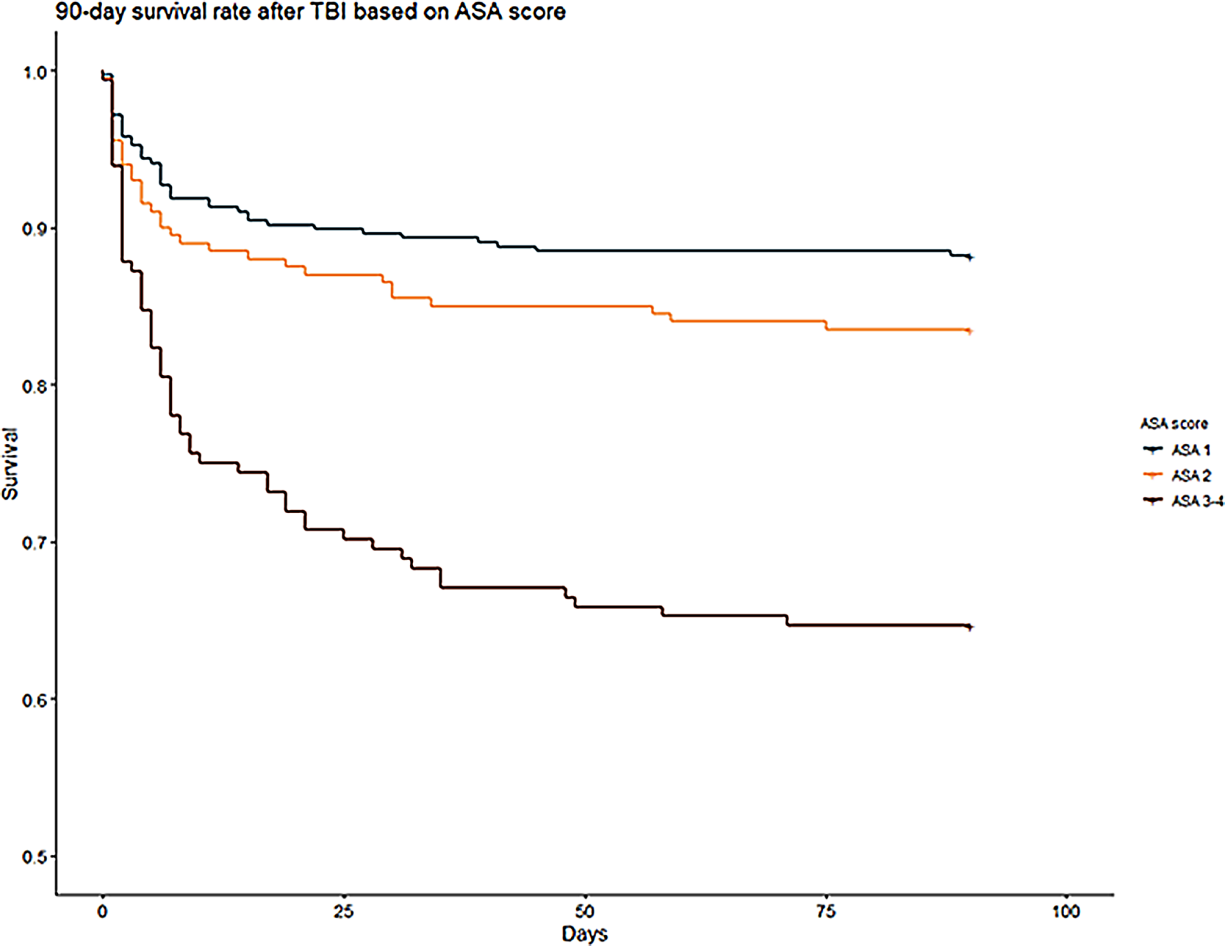
*90-day mortality and ASA score*. Kaplan-Meier survival curves tracking the 90-day survival rates, grouped according ASA scores. Three distinct survival trajectories are plotted, with the blue line representing patients with an ASA score of 1, indicating a generally healthy patient group; the orange line for patients with an ASA score of 2, signifying patients with mild systemic disease; and the red line for patients with ASA scores of 3–4, representing patients with severe systemic disease. The x-axis measures the time in days since the TBI event, while the y-axis displays the probability of survival. ASA: American Society of Anesthesiologists Classification

ASA score, GCS, TRISS and NISS were independently predictors of 90-day mortality (Table 4; Fig. 4). Hypotension, but not hypoxia, and only bilateral abnormal pupillary reaction to light, not unilateral was predictive of mortality.

**Table 4:** 90-day mortality

Parameters	*p*-value	Nagelkerke pseudo *R*^2^	OR	AUC	AIC	*n*
Pre-admission						
Age	< 0.001 *	0.10	1.04 (1.03–1.05)	0.679	647	720
Gender (Female)	0.201		1.31 (0.86–1.98)	0.527	690	720
ASA score		0.09	0 (0–0)	0.653	654	720
*2*	0.105		1.5 (0.91–2.46)			
*3*	< 0.001 *		3.81 (2.4–6.1)			
*4*	< 0.001 *		10.47 (3.2-36.79)			
Polytrauma (Yes)	0.137		0.73 (0.48–1.1)	0.534	688	718
Alc (No)	0.016 *	0.02	1.7 (1.12–2.64)	0.557	650	670
**Admission**						
GCS	< 0.001 *	0.07	0.83 (0.77–0.89)	0.624	661	720
TRISS	< 0.001 *	0.21	0.97 (0.96–0.97)	0.782	357	439
Hypoxia (Yes)	0.524		1.21 (0.66–2.11)	0.514	485	511
Hypotension (Yes)	0.001 *	0.02	3.57 (1.61–7.7)	0.532	669	711
Pupils		0.19	0 (0–0)	0.697	567	686
*unilateral*	0.349		1.47 (0.62–3.12)			
*bilateral*	< 0.001 *		8.66 (5.49–13.77)			
**Radiology**						
SAH	0.811		0.95 (0.62–1.44)	0.506	586	638
EDH	0.902		1.03 (0.68–1.55)	0.503	586	638

Assessed parameters affecting 90-day mortality. Statistical significance is evaluated by p-values, with odds ratios (OR) providing the likelihood of mortality. The model’s explanatory power is quantified by Nagelkerke’s pseudo-R², and its predictive accuracy is indicated by AUC values. AUC: Area Under the Curve; AIC: Akaike Information Criterion; ASA: American Society of Anesthesiologists Classification; GCS: Glasgow Coma Scale; TRISS: Trauma and Injury Severity Score; Alc: Alcohol; trSAH: traumatic subarachnoid hemorrhage; EDH: epidural hematoma

## Discussion

This study aimed to investigate the prognostic impact of pre-injury health status assessed with the ASA score on long-term outcomes in patients with msTBI. Our main findings demonstrate that ASA score is independently associated to 1-year outcomes and 90-day mortality after msTBI, even when accounting for known strong predictors such as age, GCS, pupillary reactivity, and radiological classifications.

Consistent with previous research, we found age to be a significant predictor of outcome [2–5, 7, 18, 34], but ASA score contributed additional explanatory power beyond age alone. Although frailty and age are frequently correlated, they represent distinct dimensions of patient vulnerability, reflecting the important difference between chronological age and biological aging status [36]. To our knowledge, this study is the first to evaluate the ASA score’s prognostic relevance in msTBI patients. Initially developed to assess outcomes from anesthesia [37], ASA score has increasingly been correlated to many different surgical procedures [38], general trauma [21, 39] and recently complicated mTBI [24]. The predictive value of comorbidities and frailty in TBI has been studied more extensively, but with contradictory results. Some studies have found a strong correlation between mortality and comorbidities, especially long-term mortality [19, 40, 41], whereas other did not [19, 41, 42], especially short-term mortality. How comorbidity and frailty is measured varies between the studies, a recognized weakness in the field [41]. The Charlson Comorbidity Index (CCI) evaluates patient status based on age and 17 predefined comorbidities [43]. While the CCI benefits from requiring only patient chart review, it is limited by its focus on comorbidities without consideration of frailty. Frailty assessment tools, such as the Clinical Frailty Scale (CFS) [44], provide comprehensive evaluation of functional frailty but necessitate both patient interviews and specialized training for accurate implementation [45, 46]. The ASA score can be rapidly determined using readily available chart data and uniquely encompasses both comorbidity burden [39] and functional frailty assessment [37] which makes it a promising tool in the management of TBI patients.

The current predictive models demonstrate a modest explanatory power, accounting for approximately 35% when relying on admission variables alone [3, 8] with an additional 5% improvement when incorporating data available during the critical care phase [47]. Although the 3% increase in explanatory power achieved by or IMPACT model + ASA might appear minor, it actually represents a relative improvement of 9.3%, aligning with similar advancements reported in other studies [48–50]. As predictive models grow more sophisticated, identifying variables that meaningfully enhance performance becomes increasingly difficult. Given the ease of obtaining the ASA score, this enhancement could lead to better risk stratification and more informed clinical decision-making, potentially improving patient outcomes: It is also a convenient tool for research, as it doesn’t require any information outside the patient’s chart and can therefore be obtained retrospectively. Comparing the significance of ASA score with other frailty and comorbidity indices is needed to assess its broader clinical implications.

TRISS was more associated with mortality than to long-term functional outcome in our study. The impact of injury severity seems to be correlated to short term outcome while other factors might shape the trajectory of survivors [41, 51]. The strong predictive value of TRISS suggests that extracranial injuries significantly contribute to global outcomes. TBI is a well-recognized risk factor in polytrauma [52], but polytrauma should maybe be more recognized as a risk factor in TBI as well. Prognostic models that focus on intracranial pathology and physiology may overlook the cumulative burden of polytrauma [11] and could be enhanced by better incorporating extracranial trauma severity and the cumulative effects of polytrauma.

Our study is not without its limitations. As a single center study in a level I hospital in Sweden, our findings may not be fully generalizable to other settings. The retrospective design restricted our analysis to available data, leading to some missing values addressed through imputation, introducing information- and representation bias. We tried to mitigate this by averaging the imputations over seven iterations, with each imputation model constructed to include variables that were predictors of both the presence of missing data and the outcome, thereby adhering to the Missing at Random (MAR) assumption, to produce more solid estimates. Thus, while imputation helped maintain the robustness of our dataset, it is important to interpret these results with caution, acknowledging the inherent limitations of retrospective data collection and the assumptions underlying the imputation techniques used. The retrospective design also introduces a potential treatment bias, as it is possible that patients with high ASA will not have their treatment escalated and care might be withheld or withdrawn due to severe comorbidities. However, our experience is that very few patients are admitted to our intensive care units unless they are deemed to have salvageable injuries and a potential for increased life-quality following their care. In conclusion, this is to the best of our knowledge the first study to analyze the added predictive value of ASA score on msTBI. The study found that pre-injury health measured by ASA score has a strong independent association to outcome and mortality and adding ASA to our IMPACT model led to a small but statistically significant improvement. Future research is warranted to confirm our findings, and the role ASA may play in comparisons of other frailty and morbidity scores in TBI research.

## Electronic supplementary material

Below is the link to the electronic supplementary material.

Supplementary Material 1

Supplementary Material 2

## Data Availability

No datasets were generated or analysed during the current study.

## Abbreviations

AIC: Akaike information criterion
AIS: Abbreviated Injury Score
ASA: American Society of Anesthesiologists
AUC: Area under the curve
CRASH: Corticosteroid Randomization After Significant Head Injury
EDH: Epidural hematoma
GCS: Glasgow Coma Scale
GOS: Glasgow Outcome Scale
IMPACT: International Mission for Prognosis and Analysis of Clinical Trials in TBI
ISS: Injury severity score
MAR: Missing at Random
msTBI: Moderate-to-severe TBI
NISS: New injury severity score
ROC: Receiver Operating Characteristic
RTS: Revised Trauma Score
SBP: Systolic blood pressure
TBI: Traumatic Brain Injury
TRISS: Trauma and Injury Severity Score
trSAH: Traumatic subarachnoid hemorrhage

## References

[1] Hyder AA, Wunderlich CA, Puvanachandra P, Gururaj G, Kobusingye OC. The impact of traumatic brain injuries: a global perspective. NeuroRehabilitation. 2007;22(5):341–53. 18162698

[2] Kirkman MA, Jenks T, Bouamra O, Edwards A, Yates D, Wilson MH. Increased mortality associated with cerebral contusions following trauma in the elderly: bad patients or bad management? J Neurotrauma. 2013;30(16):1385–90. 23441674 10.1089/neu.2013.2881

[3] Maas AI, Lingsma HF, Roozenbeek B. Predicting outcome after traumatic brain injury. Handb Clin Neurol. 2015;128:455–74. 25701901 10.1016/B978-0-444-63521-1.00029-7

[4] Collaborators MCT, Perel P, Arango M, Clayton T, Edwards P, Komolafe E, et al. Predicting outcome after traumatic brain injury: practical prognostic models based on large cohort of international patients. BMJ. 2008;336(7641):425–9. 18270239 10.1136/bmj.39461.643438.25 PMC2249681

[5] Maas AI, Marmarou A, Murray GD, Teasdale SG, Steyerberg EW. Prognosis and clinical trial design in traumatic brain injury: the IMPACT study. J Neurotrauma. 2007;24(2):232–8. 17375987 10.1089/neu.2006.0024

[6] Marmarou A, Lu J, Butcher I, McHugh GS, Mushkudiani NA, Murray GD, et al. IMPACT database of traumatic brain injury: design and description. J Neurotrauma. 2007;24(2):239–50. 17375988 10.1089/neu.2006.0036

[7] Helmrich I, Czeiter E, Amrein K, Buki A, Lingsma HF, Menon DK, et al. Incremental prognostic value of acute serum biomarkers for functional outcome after traumatic brain injury (CENTER-TBI): an observational cohort study. Lancet Neurol. 2022;21(9):792–802. 35963262 10.1016/S1474-4422(22)00218-6

[8] Thelin E, Al Nimer F, Frostell A, Zetterberg H, Blennow K, Nystrom H, et al. A serum protein Biomarker Panel improves Outcome Prediction in Human Traumatic Brain Injury. J Neurotrauma. 2019;36(20):2850–62. 31072225 10.1089/neu.2019.6375 PMC6761606

[9] Thelin EP, Nelson DW, Vehvilainen J, Nystrom H, Kivisaari R, Siironen J, et al. Evaluation of novel computerized tomography scoring systems in human traumatic brain injury: an observational, multicenter study. PLoS Med. 2017;14(8):e1002368. 28771476 10.1371/journal.pmed.1002368 PMC5542385

[10] Tjerkaski J, Nystrom H, Raj R, Lindblad C, Bellander BM, Nelson DW, et al. Extended Analysis of Axonal Injuries Detected Using Magnetic Resonance Imaging in critically ill traumatic brain Injury patients. J Neurotrauma. 2022;39(1–2):58–66. 34806407 10.1089/neu.2021.0159 PMC8785713

[11] Eagle SR, Nwachuku E, Elmer J, Deng H, Okonkwo DO, Pease M. Performance of CRASH and IMPACT Prognostic models for traumatic Brain Injury at 12 and 24 months Post-injury. Neurotrauma Rep. 2023;4(1):118–23. 36895818 10.1089/neur.2022.0082 PMC9989509

[12] Akerlund CAI, Holst A, Stocchetti N, Steyerberg EW, Menon DK, Ercole A, et al. Clustering identifies endotypes of traumatic brain injury in an intensive care cohort: a CENTER-TBI study. Crit Care. 2022;26(1):228. 35897070 10.1186/s13054-022-04079-w PMC9327174

[13] Penn-Barwell JG, Bishop JRB, Midwinter MJ. Refining the Trauma and Injury Severity score (TRISS) to measure the performance of the UK Combat Casualty Care System. Mil Med. 2018;183(9–10):e442–7. 29365167 10.1093/milmed/usx039

[14] Boyd CR, Tolson MA, Copes WS. Evaluating trauma care: the TRISS method. Trauma score and the Injury Severity score. J Trauma. 1987;27(4):370–8. 3106646

[15] Domingues CA, Coimbra R, Poggetti RS, Nogueira LS, de Sousa RMC. New Trauma and Injury Severity score (TRISS) adjustments for survival prediction. World J Emerg Surg. 2018;13:12. 29541155 10.1186/s13017-018-0171-8 PMC5840784

[16] Kiwanuka O, Lassaren P, Hanell A, Bostrom L, Thelin EP. ASA-score is associated with 90-day mortality after complicated mild traumatic brain injury - a retrospective cohort study. Acta Neurochir (Wien). 2024;166(1):363. 39259285 10.1007/s00701-024-06247-z PMC11390782

[17] Wong GK, Teoh J, Yeung J, Chan E, Siu E, Woo P, et al. Outcomes of traumatic brain injury in Hong Kong: validation with the TRISS, CRASH, and IMPACT models. J Clin Neurosci. 2013;20(12):1693–6. 23993210 10.1016/j.jocn.2012.12.032

[18] Skaansar O, Tverdal C, Ronning PA, Skogen K, Brommeland T, Roise O, et al. Traumatic brain injury-the effects of patient age on treatment intensity and mortality. BMC Neurol. 2020;20(1):376. 33069218 10.1186/s12883-020-01943-6 PMC7568018

[19] Xiong C, Hanafy S, Chan V, Hu ZJ, Sutton M, Escobar M, et al. Comorbidity in adults with traumatic brain injury and all-cause mortality: a systematic review. BMJ Open. 2019;9(11):e029072. 31699721 10.1136/bmjopen-2019-029072 PMC6858248

[20] Fitz-Henry J. The ASA classification and peri-operative risk. Ann R Coll Surg Engl. 2011;93(3):185–7. 21477427 10.1308/147870811X565070a PMC3348554

[21] Skaga NO, Eken T, Sovik S, Jones JM, Steen PA. Pre-injury ASA physical status classification is an independent predictor of mortality after trauma. J Trauma. 2007;63(5):972–8. 17993938 10.1097/TA.0b013e31804a571c

[22] Kuza CM, Matsushima K, Mack WJ, Pham C, Hourany T, Lee J, et al. The role of the American Society of anesthesiologists physical status classification in predicting trauma mortality and outcomes. Am J Surg. 2019;218(6):1143–51. 31575418 10.1016/j.amjsurg.2019.09.019 PMC8421011

[23] Kiwanuka O, Lassaren P, Thelin EP, Hanell A, Sandblom G, Fagerdahl A, et al. Long-term health-related quality of life after trauma with and without traumatic brain injury: a prospective cohort study. Sci Rep. 2023;13(1):2986. 36805021 10.1038/s41598-023-30082-4 PMC9941121

[24] Kiwanuka OL, Hånell P, Boström A, Thelin L. EP. ASA–score is associated with 90–day mortality after complicated mild traumatic brain injury– a retrospective cohort study. Acta Neurochir (Wien). 2024 Sep 11;166(1):363. 10.1007/s00701-024-06247-z PMC11390782 39259285

[25] Ringdal KG, Coats TJ, Lefering R, Di Bartolomeo S, Steen PA, Roise O, et al. The Utstein template for uniform reporting of data following major trauma: a joint revision by SCANTEM, TARN, DGU-TR and RITG. Scand J Trauma Resusc Emerg Med. 2008;16:7. 18957069 10.1186/1757-7241-16-7 PMC2568949

[26] Abbreviated IS. 2005 Update 2008. Gennarelli T WE, editor. Chicago, USA: Association for the Advancement of Automotive Medicine; 2016.

[27] Rating the severity. Of tissue damage. I. The abbreviated scale. JAMA. 1971;215(2):277–80. 5107365 10.1001/jama.1971.03180150059012

[28] Loftis KL, Price J, Gillich PJ. Evolution of the abbreviated Injury Scale: 1990–2015. Traffic Inj Prev. 2018;19(sup2):S109–13. 30543458 10.1080/15389588.2018.1512747

[29] Gross T, Schuepp M, Attenberger C, Pargger H, Amsler F. Outcome in polytraumatized patients with and without brain injury. Acta Anaesthesiol Scand. 2012;56(9):1163–74. 22735047 10.1111/j.1399-6576.2012.02724.x

[30] Champion HR, Sacco WJ, Carnazzo AJ, Copes W, Fouty WJ. Trauma score. Crit Care Med. 1981;9(9):672–6. 7273818 10.1097/00003246-198109000-00015

[31] Jennett B, Bond M. Assessment of outcome after severe brain damage. Lancet. 1975;1(7905):480–4. 46957 10.1016/s0140-6736(75)92830-5

[32] Team RC. R. 2022.

[33] Marshall A, Altman DG, Royston P, Holder RL. Comparison of techniques for handling missing covariate data within prognostic modelling studies: a simulation study. BMC Med Res Methodol. 2010;10:7. 20085642 10.1186/1471-2288-10-7 PMC2824146

[34] Murray GD, Butcher I, McHugh GS, Lu J, Mushkudiani NA, Maas AI, et al. Multivariable prognostic analysis in traumatic brain injury: results from the IMPACT study. J Neurotrauma. 2007;24(2):329–37. 17375997 10.1089/neu.2006.0035

[35] Panken AM, Heymans MW. A simple pooling method for variable selection in multiply imputed datasets outperformed complex methods. BMC Med Res Methodol. 2022;22(1):214. 35927610 10.1186/s12874-022-01693-8 PMC9351113

[36] Xue QL. The frailty syndrome: definition and natural history. Clin Geriatr Med. 2011;27(1):1–15. 21093718 10.1016/j.cger.2010.08.009 PMC3028599

[37] Mayhew D, Mendonca V, Murthy BVS. A review of ASA physical status - historical perspectives and modern developments. Anaesthesia. 2019;74(3):373–9. 30648259 10.1111/anae.14569

[38] Hackett NJ, De Oliveira GS, Jain UK, Kim JY. ASA class is a reliable independent predictor of medical complications and mortality following surgery. Int J Surg. 2015;18:184–90. 25937154 10.1016/j.ijsu.2015.04.079

[39] Ringdal KG, Skaga NO, Steen PA, Hestnes M, Laake P, Jones JM, et al. Classification of comorbidity in trauma: the reliability of pre-injury ASA physical status classification. Injury. 2013;44(1):29–35. 22277107 10.1016/j.injury.2011.12.024

[40] Dell KC, Grossner EC, Staph J, Schatz P, Hillary FG. A Population-based study of Pre-existing Health conditions in Traumatic Brain Injury. Neurotrauma Rep. 2021;2(1):255–69. 34223556 10.1089/neur.2020.0065 PMC8244518

[41] Roohollahi F, Molavi S, Mohammadi M, Mohamadi M, Mohammadi A, Kankam SB, et al. Prognostic value of Frailty for Outcome following traumatic Brain Injury: a systematic review and Meta-analysis. J Neurotrauma. 2024;41(3–4):331–48. 37416987 10.1089/neu.2023.0176

[42] Orso D, Furlanis G, Romanelli A, Gheller F, Tecchiolli M, Cominotto F. Risk factors analysis for 90-Day mortality of adult patients with mild traumatic brain Injury in an Italian Emergency Department. Geriatr (Basel). 2024;9(2). 10.3390/geriatrics9020023 PMC10961819 38525740

[43] Charlson ME, Pompei P, Ales KL, MacKenzie CR. A new method of classifying prognostic comorbidity in longitudinal studies: development and validation. J Chronic Dis. 1987;40(5):373–83. 3558716 10.1016/0021-9681(87)90171-8

[44] Church S, Rogers E, Rockwood K, Theou O. A scoping review of the clinical Frailty Scale. BMC Geriatr. 2020;20(1):393. 33028215 10.1186/s12877-020-01801-7 PMC7540438

[45] Albrecht R, Espejo T, Riedel HB, Nissen SK, Banerjee J, Conroy SP, et al. Clinical Frailty Scale at presentation to the emergency department: interrater reliability and use of algorithm-assisted assessment. Eur Geriatr Med. 2024;15(1):105–13. 37971677 10.1007/s41999-023-00890-y PMC10876739

[46] van der Burgh R, Wijnen N, Visscher M, de Groot B, Lucke J. The feasibility and acceptability of frailty screening tools in the Emergency Department and the additional value of clinical judgment for frailty detection. Eur J Emerg Med. 2022;29(4):301–3. 35773203 10.1097/MEJ.0000000000000910

[47] Bhattacharyay S, Caruso PF, Akerlund C, Wilson L, Stevens RD, Menon DK, et al. Mining the contribution of intensive care clinical course to outcome after traumatic brain injury. NPJ Digit Med. 2023;6(1):154. 37604980 10.1038/s41746-023-00895-8 PMC10442346

[48] Nelson DW, Rudehill A, MacCallum RM, Holst A, Wanecek M, Weitzberg E, et al. Multivariate outcome prediction in traumatic brain injury with focus on laboratory values. J Neurotrauma. 2012;29(17):2613–24. 22994879 10.1089/neu.2012.2468

[49] Thelin EP, Jeppsson E, Frostell A, Svensson M, Mondello S, Bellander BM, et al. Utility of neuron-specific enolase in traumatic brain injury; relations to S100B levels, outcome, and extracranial injury severity. Crit Care. 2016;20:285. 27604350 10.1186/s13054-016-1450-y PMC5015335

[50] Zeiler FA, Ercole A, Beqiri E, Cabeleira M, Thelin EP, Stocchetti N, et al. Association between Cerebrovascular Reactivity Monitoring and Mortality is preserved when adjusting for baseline admission characteristics in Adult Traumatic Brain Injury: A CENTER-TBI Study. J Neurotrauma. 2020;37(10):1233–41. 31760893 10.1089/neu.2019.6808 PMC7232651

[51] Galimberti S, Graziano F, Maas AIR, Isernia G, Lecky F, Jain S, et al. Effect of frailty on 6-month outcome after traumatic brain injury: a multicentre cohort study with external validation. Lancet Neurol. 2022;21(2):153–62. 35065038 10.1016/S1474-4422(21)00374-4

[52] Niemeyer M, Jochems D, Houwert RM, van Es MA, Leenen L, van Wessem K. Mortality in polytrauma patients with moderate to severe TBI on par with isolated TBI patients: TBI as last frontier in polytrauma patients. Injury. 2022;53(4):1443–8. 35067344 10.1016/j.injury.2022.01.009

