# Supplementary material for: ASA score is an independent predictor of 1-year outcome after moderate-to-severe traumatic brain injury
Source: Scand J Trauma Resusc Emerg Med. 2025 Feb 6;33:25. doi: 10.1186/s13049-025-01338-x (PMC11804083; doi:10.1186/s13049-025-01338-x)
Supplement: Supplementary file 1 — Supplementary Material 1 [file 13049_2025_1338_MOESM1_ESM.pdf]

Supplementary figure 1: inclusion flowchart

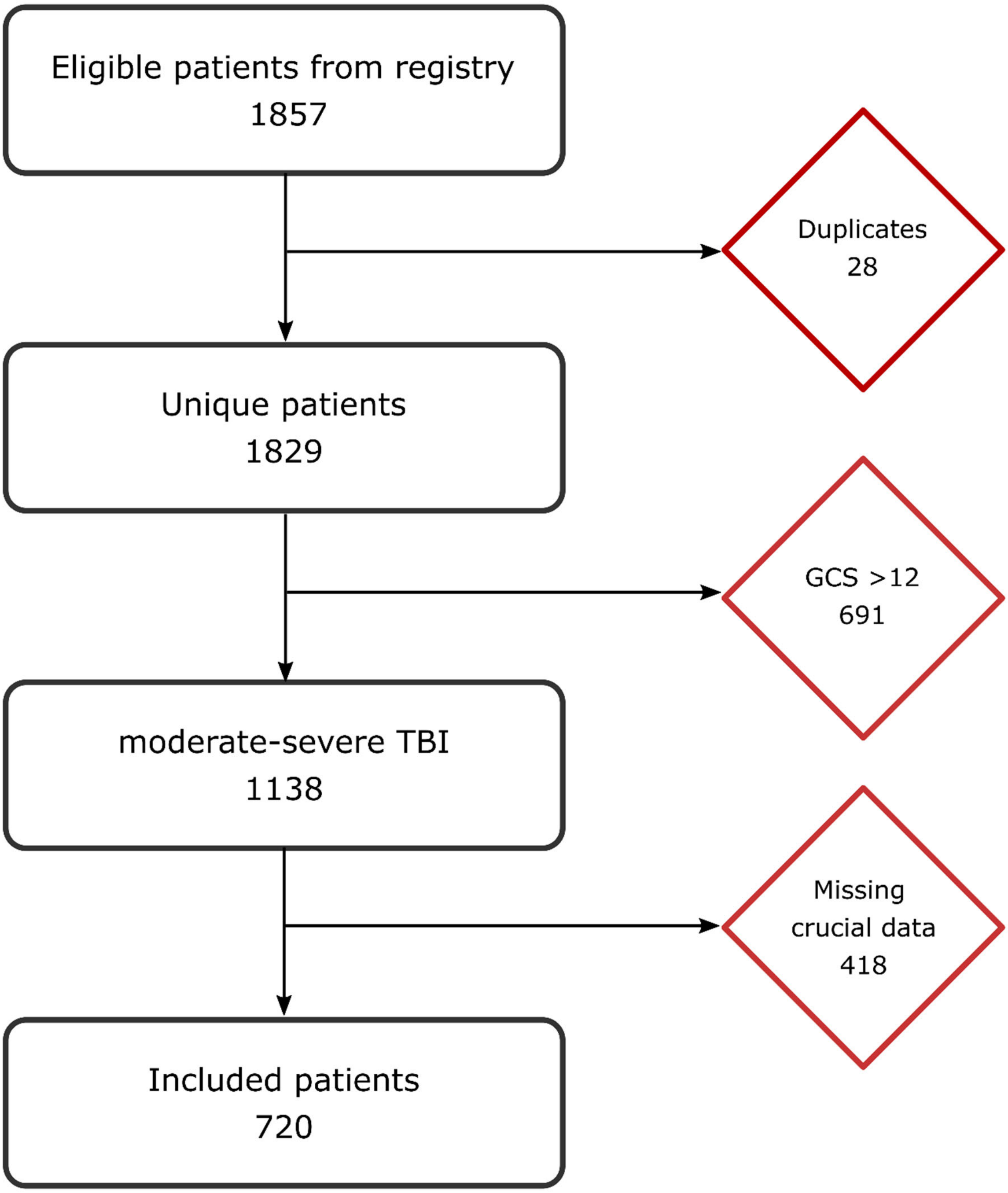

Supplementary figure 1: Flowchart of the selection process of patients. After the removal of 28 duplicates, 691 with mild TBI, 417 missing ASA scores and 1 Marshall classification, a total of 720 patients met the inclusion criteria for the study.
