# Supplementary material for: ASA score is an independent predictor of 1-year outcome after moderate-to-severe traumatic brain injury
Source: Scand J Trauma Resusc Emerg Med. 2025 Feb 6;33:25. doi: 10.1186/s13049-025-01338-x (PMC11804083; doi:10.1186/s13049-025-01338-x)
Supplement: Supplementary file 2 — Supplementary Material 2 [file 13049_2025_1338_MOESM2_ESM.pdf]

Supplementary table 1: TRISS formula

**Probability of survival (Ps)**	**P(s) = 1/ (1+ e^{-b})**	
**b**	b = b_0 + b_1 x RTS + b_2 x ISS + b_3 x age index,	
	blunt	*penetrating*
b0	-0,4499	-2,5355
b1	0,8085	0,9934
b2	-0,0835	-0,0651
b3	-1,743	-1,136
**Revised Trauma Score(RTS)**	RTS = 0.9386 (GCSc) + 0.7326 (SBPc) + 0.2908 (RRc)	
GCS	*GCSc*	
13-15	4	
9-12	3	
6-8	2	
4-5	1	
3	0	
SBP	*SBPc*	
>89	4	
76-89	3	
50-75	2	
1-49	1	
0	0	
RR	*RRc*	
10-29	4	
>29	3	
6-9	2	
1-5	1	
0	0	
**ISS**	(1st AIS score)^2+(2nd AIS score)^2+(3rd AIS score)^2	
**Age-index**		
Age <55	0	
Age ≥55	1	

Supplementary table 1: TRISS is calculated by combining RTS, ISS, and an age index, weighted according to the type of injury (blunt or penetrating). **TRISS**: Trauma and Injury Severity Score; **RTS**: Revised Trauma Score; **ISS**: Injury Severity Score; **GCS**: Glasgow Coma Scale.
